# Case Report: Microincision Vitreous Surgery Induces Bleb Failure in Eyes With Functional Filtering Bleb

**DOI:** 10.3389/fmed.2022.847660

**Published:** 2022-02-21

**Authors:** Satomi Oogi, Shunsuke Nakakura, Ryo Asaoka, Etsuko Terao, Saki Dote, Kanae Matsuya, Yui Kimura

**Affiliations:** ^1^Department of Ophthalmology, Saneikai Tsukazaki Hospital, Himeji, Japan; ^2^Department of Ophthalmology, Seirei Hamamatsu General Hospital, Hamamatsu, Japan; ^3^Seirei Christopher University, Hamamatsu, Japan; ^4^Nanovision Research Division, Research Institute of Electronics, Shizuoka University, Hamamatsu, Japan; ^5^The Graduate School for the Creation of New Photonics Industries, Hamamatsu, Japan; ^6^Department of Ophthalmology, Graduate School of Medicine and Faculty of Medicine, The University of Tokyo, Tokyo, Japan

**Keywords:** glaucoma, trabeculectomy, Ex-PRESS, microincision vitreous surgery, IOP (intraocular pressure)

## Abstract

**Purpose:**

To investigate the effect of microincision vitreous surgery (MIVS) on intraocular pressure (IOP) control in glaucomatous eyes with functional filtering bleb.

**Methods:**

We enrolled 18 patients (15 males; median age, 73 years) who previously had filtering surgery and underwent MIVS with functional filtering bleb. Kaplan–Meier method was used to calculate the survival rate with defined the failure as when more number of preoperative antiglaucoma medication was started or additional glaucoma surgery including bleb revisions were performed, and IOP increase of 20% (criteria 1) and 30% (criteria 2) from preoperative levels after 2 weeks of MIVS.

**Results:**

The median follow-up duration was 970 days. Preoperative IOP was 13.3 ± 3.8 mmHg (mean ± SD). Postoperative IOP were 14.7 ± 4.9 (*P*=0.365), 15.2 ± 3.5 (*P*=0.137), 16.4 ± 5.6 (*P* = 0.073), 17.6 ± 6.1(*P* = 0.020), and 14.5 ± 4.0 (*P* = 0.402) mmHg at 3, 6, 12, and 15 months and final visit, respectively (compared to preoperative IOP). The number of antiglaucoma medications was a median of 1.0 (range 0–4) preoperatively and 0 (0–4) at the final visit (*P* = 0.238). The survival rates were 55%/61% at 3 months, 50%/61% at 6 months, and 38%/55% at 12 months with criteria 1 and 2, respectively. Four eyes (22%) received additional glaucoma surgery during follow-up.

**Conclusion:**

After several months of MIVS, IOP was likely to increase. We should focus on IOP control by conducting long-term follow-ups.

## Introduction

Microincision vitreous surgery (MIVS) using 23-G, 25-G, and 27-G systems is much less invasive compared with vitreous surgery using a 20-G system and currently performed worldwide. Therefore, MIVS may be beneficial for patients with glaucoma, especially for those who have undergone filtering surgery. Meanwhile, it has been suggested that the removal of the vitreous itself, regardless of the mode of surgery (MIVS or 20-G system), may result in the increase in intraocular pressure (IOP) (1, 2) and glaucoma prevalence (3–5). The entire mechanism underlying chronic IOP increase after vitrectomy is unknown; however, it may be attributed to the increase in the partial pressure of oxygen (pO_2_) (3, 4, 6). pO_2_ is the highest near the retinal surface, low in the anterior vitreous chamber, and lowest at the center of the lens (3). However, after cataract surgery and vitrectomy, pO_2_ level increased in the vitreous cavity and anterior chamber angle, leading to oxidative damage of the trabecular meshwork cells, thus decreasing outflow facility (6). This implies that in a patient with filtering bleb after vitrectomy, careful considerations regarding IOP must be made even when performing MIVS. To date, there are only two reports that have examined postoperative IOP control in patients with bleb who had undergone MIVS (7, 8). Both studies showed that there were some patients who needed more antiglaucoma medications and additional glaucoma surgeries after MIVS, although mean IOP during follow-up had not significantly changed (7, 8). However, they added the effect of additional glaucoma surgery in their analysis and the follow-up duration was relatively short (11.5 and 16.0 months). Therefore, the actual effect of MIVS on IOP control in patients with functional filtering bleb remains to be fully elucidated. This study aims to evaluate the effect of MIVS on IOP control in patients that have functional filtering bleb.

## Materials and Methods

This retrospective study received approval from the Institutional Review Board of Saneikai Tsukazaki Hospital, Himeji, Japan (No: 211031) and was conducted according to the tenets of the Declaration of Helsinki. We reviewed the medical records of Saneikai Tsukazaki Hospital to enroll patients who had undergone MIVS after filtering surgery [trabeculectomy or EX-PRESS shunt (Alcon Laboratories, Fort Worth, TX, USA)] between January 2014 and December 2020.

The inclusion criteria were as follows: (1) patients with glaucoma and functional filtering bleb (functional bleb was defined as an IOP of ≤ 21 mmHg before MIVS and >20% reduction compared to that before filtering surgery), (2) a minimum follow-up duration of at least 6 months after MIVS, and (3) patients without additional MIVS or those with endophthalmitis caused by bleb infections. Among the 30 patients enrolled during the specified period, 18 patients who met the selection criteria were included. Furthermore, we subcategorized patients who underwent MIVS into full vitrectomy and core vitrectomy only groups.

Most vitreous surgeons once quite the glaucoma medications after MIVS because of increase in the use of postoperative medications (such as antibiotics, nonsteroidal anti-inflammatory drugs, and steroids) or the presence of small leakages from wounds caused by the use of trocars during vitrectomy. IOP increases after MIVS were dealt according to each surgeon's choice (e.g., reinitiating antiglaucoma medication or performing bleb revision or bleb needling). Therefore, we defined failure as the condition in which a high number of preoperative antiglaucoma medications or additional glaucoma surgery including bleb revisions was required and when IOP increases of 20% (criteria 1) or 30% (criteria 2) from preoperative levels were observed at two consecutive follow-ups performed after 2 weeks of MIVS.

Statistical analyses were performed using BellCurve for Excel (Social Survey Research Information Co., Tokyo, Japan) and the statistical program R software (version 3.6.1, http://www.rproject.org/). The Shapiro–Wilk test was used to verify data distribution normality. IOPs were presented as mean ± standard deviation and compared using the paired *t*-test. The number of glaucoma medications was presented as median (min–max) and compared using the Mann–Whitney *U*-test. *P* < 0.05% was considered statistically significant. Multivariate Cox proportional hazards regression analysis was conducted for MIVS type (full vitrectomy/core vitrectomy), glaucoma type, previous filtering surgery type (trabeculectomy/EX-PRESS shunt), preoperative IOP, and preoperative antiglaucoma medication number to identify risk factors for surgical failure. All IOPs were measured using IcareTA01i (Icare Finland Oy, Helsinki, Finland) to prevent discrepancies in the tonometery performed during follow-ups. Central corneal thickness before MIVS was measured using a specular microscope (Konan Noncon ROBO SP-6000, Konan Medical Inc., Hyogo, Japan).

## Results

Among the 18 included patients, 15 were males (83%) accounting for 12 (66%) right eyes. The median age of the patients was 73 (quantile; 68–78). The types of glaucoma were primary open-angle glaucoma (*N* = 8, 44%), neovascular glaucoma (*N* = 4, 22%), exfoliation glaucoma (*N* = 4, 22%), and secondary glaucoma (*N* = 2, 11%). The median follow-up duration after MIVS was 970 days (506–1,474) and that after filtering surgery (performed before MIVS) was 419 days (165–900). Eleven and 7 eyes underwent previous filtering surgery by trabeculectomy and EX-PRESS shunt, respectively. The reason for performing MIVS was transconjunctival intrascleral fixation of an intraocular lens in seven eyes, vitreous hemorrhage in five eyes, macular hole in two eyes, macular edema in two eyes, and proliferative tissue removal in two eyes. Central corneal thickness was 517 ± 47 (range: 448–615) μm.

Change in IOP with additional glaucoma medication and interventions, number of glaucoma medications are summarized in Table 1. IOPs tended to gradually increase post-MIVS. IOP at 15 months post-MIVS was significantly higher than that of pre-MIVS (17.6 mmHg vs. 13.3 mmHg, *P* = 0.020). However, no significant difference was found between IOPs pre-MIVS and those at 1, 2, 3, 6, 9, and 12 months post-MIVS. The number of medications was lesser at 3 months post-MIVS compared to that of pre-MIVS. However, compared with pre-MIVS, the number of medications at 6 months post-MIVS was not significantly different from that observed between 6 months post-MIVS and final visit. Before the final visit, two eyes underwent additional revision surgery and bleb needling (67 and 336 days post-MIVS, respectively), one eye underwent Ahmed glaucoma valve implantation (838 days post-MIVS), and one eye underwent EX-PRESS shunt operation (1,234 days post-MIVS). None of the eyes satisfied the criteria of failure when defined as more number of preoperative anti-glaucoma medication after 2 weeks of MIVS, with both criteria.

**Table 1:** Changes in IOPs including additional intervention and the number of antiglaucoma medications.

	**Number**	**IOP**	***P*** **-value**	**Number of glaucoma medications**	***P*** **-value**
Pre-MIVS	18	13.3 ± 3.8 (3–19)		1.0 (0–4)	
1 month	18	15.0 ± 4.6 (3–26)	0.262	0 (0–2)	0.001
2 months	16	15.1 ± 5.3 (3–23)	0.262	0 (0–2)	0.003
3 months	17	14.7 ± 4.9 (6–25)	0.365	0 (0–3)	0.027
6 months	18	15.2 ± 3.5 (7–21)	0.137	0 (0–3)	0.161
9 months	17	14.7 ± 5.3 (4–25)	0.407	0 (0–3)	0.147
12 months	15	16.4 ± 5.6 (6–32)	0.073	0 (0–3)	0.266
15 months	15	17.6 ± 6.1 (12–30)	0.020	0 (0–3)	0.266
18 months	11	15.2 ± 4.4 (9–25)	0.203	0 (0–3)	0.356
21 months	11	15.3 ± 4.1 (9–25)	0.230	0 (0–3)	0.474
24 months	11	17.7 ± 4.1 (7–30)	0.041	0 (0–3)	0.542
Final visit	18	14.5 ± 4.0 (8–22)	0.402	0 (0–4)	0.238

*Each P value was compared pre-MIVS, and IOPs at each time point were calculated using paired t-test and Mann–Whitney U-test. The number of glaucoma medications was expressed as median with range*.

**Figure 1:**
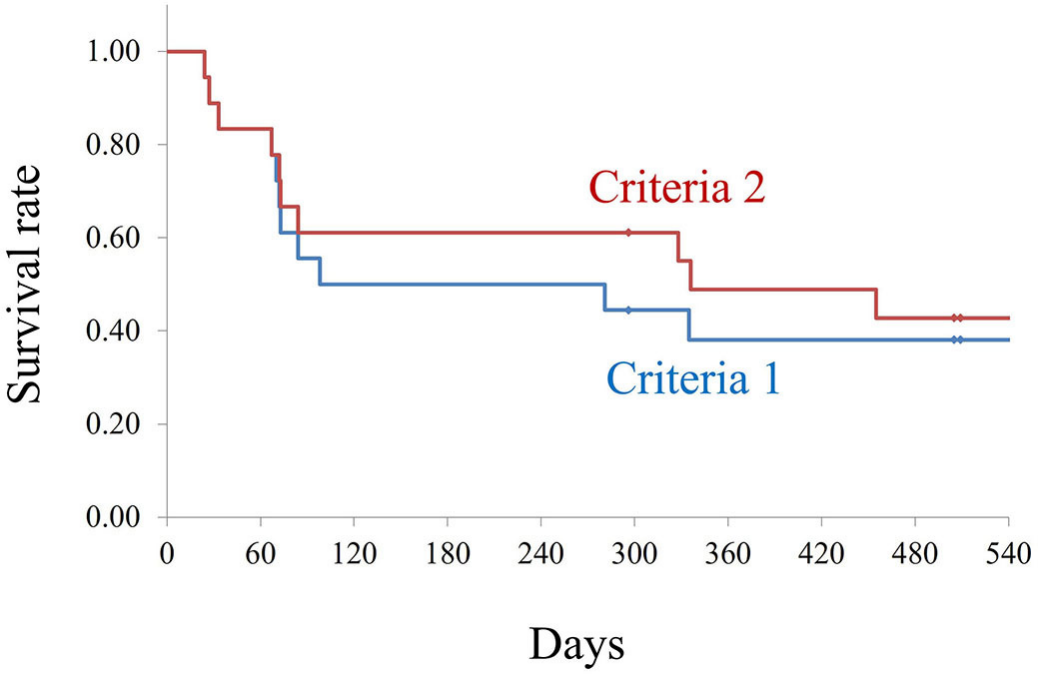
Kaplan–Meier survival analysis for both the criteria. Criteria 1: Number of antiglaucoma medications similar or less than the preoperative number, no additional glaucoma surgery, and introcular pressure (IOP) increase of less than 20% compared with pre-microincision vitreous surgery (pre-MIVS) IOP. Criteria 2: Number of antiglaucoma medications similar or less than the preoperative number, no additional glaucoma surgery, and IOP increase of <30% compared with pre-MIVS IOP. The log-rank test did not show any statistically significant differences between the two criteria (*P* = 0.386).

To exclude the effect of glaucoma medications and IOP after additional glaucoma surgery, we used Kaplan–Meier plots, as shown in Figure 1. The survival rates for criteria 1 were 88, 83, 55, 50, 50, 38, 38, 38, and 38% at 1, 2, 3, 6, 9, 12, 15, 18, and 24 months, respectively. The survival rates for criteria 2 were 88, 83, 61, 61, 61, 55, 42, 42, and 42% at 1, 2, 3, 6, 9, 12, 15, 18, and 24 months, respectively.

Multivariate Cox proportional hazards regression analysis revealed no significant predictive factor (Supplementary Table 1).

## Discussion

Our results revealed that the risk of IOP increases after MIVS in patients with functional blebs. The survival rate decreased to 50% and 61% at 6 months. Two previous studies concluded that MIVS does not adversely affect bleb function (7, 8). To elaborate, Kunikakta et al. reported 15 cases of MIVS performed after filtering surgery; preoperative IOP was 11.9 mmHg with the use of 1.2 glaucoma medications, which changed to 15.7 mmHg with the use of 1.9 glaucoma medication at the final visit (mean follow-up duration was 11.5 months), and 3 eyes needed additional trabeculectomy (7). Kim et al. also reported 11 cases in which preoperative IOP was 13.8 mmHg, which increased to 15.8 mmHg at the final visit (mean follow-up duration was 16.0 months), and 1 eye needed additional glaucoma surgery (8).

IOPs were not statistically significant during the follow-up period (*P* = 0.20, 0.758, respectively) (7, 8). However, they added the effect of additional glaucoma surgery in their analysis and their follow-up duration was shorter than that in our study; they also did not perform life survival analysis. Therefore, our results shed light on the actual IOP control after MIVS.

Toyokawa et al. showed no significant differences in the incidence of IOP increase (>4 mmHg) after MIVS among the patients with different types of vitreoretinal diseases (mean follow-up duration of 47.8 months) (1). However, Yamamoto et al. reported that IOP increases (≥ 4 mmHg) were observed only in the eyes undergoing rhegmatogenous retinal detachment surgery (full vitrectomy) and not in those undergoing epiretinal membrane and macular hole surgeries (core vitrectomy) (mean follow-up of 23.9 months) (2). Thus, we investigated the effect of MIVS type (full vitrectomy/core vitrectomy) using multivariate Cox proportional hazards regression analysis, but it was not identified as a signficant factor. Studies investigating the effect of removing the vitreous cavity on IOP increases are needed. A previous review by Rossi reported that the causes of chronic IOP increases after vitrectomy are angle synechia (chronic inflammation in the anterior chamber, intermittent closure), neovascularization (secondary to anterior segment ischemia), silicon oil glaucoma, and open-angle glaucoma (oxidative stress increases trabecular resistance) (4). We speculate that the reason for IOP increases in our cases may be a combination of both chronic inflammation and oxidative stress as described above. Therefore, we should pay attention to patients with glaucoma who have undergone MIVS and need longer follow-ups.

The first limitation of our study was the inclusion of a small number of cases because of the single-center design and the lack of a control group. Nonetheless, our sample size is larger than that of previous studies (7, 8). The second limitation is the study's retrospective nature. Furthermore, the fact that the medications were added or their use was reinitiated as per the vitreous surgeon did not lead to uniform analysis. Third, IcareTA01i has the tendency to show lower IOPs compared to Goldmann applanation tonometer (9).

In conclusion, after MIVS, we should pay attention to IOP control by conducting long-term follow-ups. Studies with larger sample sizes are needed to confirm the effect of MIVS on IOP control in patients with bleb.

## Precis

After MIVS, we should pay attention to IOP increase during long-term follow-up since MIVS may induce early bleb failure in patients with functional bleb.

## Data Availability Statement

The raw data supporting the conclusions of this article will be made available by the authors, without undue reservation.

## Ethics Statement

The studies involving human participants were reviewed and approved by IRB at Saneikai Tsukazaki Hospital. Written informed consent for participation was not required for this study in accordance with the national legislation and the institutional requirements.

## Author Contributions

SN and RA: writing and statics. All authors confirmed the submitting this article and data collected. All authors contributed to the article and approved the submitted version.

## Conflict of Interest

The authors declare that the research was conducted in the absence of any commercial or financial relationships that could be construed as a potential conflict of interest.

## Publisher's Note

All claims expressed in this article are solely those of the authors and do not necessarily represent those of their affiliated organizations, or those of the publisher, the editors and the reviewers. Any product that may be evaluated in this article, or claim that may be made by its manufacturer, is not guaranteed or endorsed by the publisher.
