# Supplementary material for: Case Report: Microincision Vitreous Surgery Induces Bleb Failure in Eyes With Functional Filtering Bleb
Source: Front Med (Lausanne). 2022 Feb 21;9:847660. doi: 10.3389/fmed.2022.847660 (PMC8899033; doi:10.3389/fmed.2022.847660)
Supplement: Supplementary file 1 [file Table_1.docx]

**Supplemental Table 1.** Results of the multivariate Cox proportional hazards regression analysis

| Criteria 1 | Beta | Upper 95% | Lower 95% | p |
| --- | --- | --- | --- | --- |
| Full vitrectomy | 1.7743 | 0.16841 | 17.766 | 0.645 |
| POAG | 0.2592 | 0.03051 | 2.282 | 0.226 |
| SG | 3.4517 | 0.14002 | 63.46 | 0.484 |
| XFG | 0.7181 | 0.06979 | 7.612 | 0.792 |
| LET | 1.2126 | 0.21663 | 5.768 | 0.894 |
| Preoperative IOP | 0.7927 | 0.6075 | 1.075 | 0.143 |
| Preoperative antiglaucoma medication number | 0.7010 | 0.40355 | 1.302 | 0.282 |
| Criteria 2 | Beta | Upper 95% | Lower 95% | p |
| Full vitrectomy | 2.752 | 0.26117 | 28.998 | 0.3995 |
| POAG | 0.1463 | 0.01354 | 1.581 | 0.1135 |
| SG | 1.1994 | 0.06642 | 21.658 | 0.902 |
| XFG | 0.6401 | 0.06695 | 6.12 | 0.6986 |
| LET | 1.2244 | 0.22543 | 6.651 | 0.8146 |
| Preoperative IOP | 0.7991 | 0.59712 | 1.069 | 0.1313 |
| Preoperative antiglaucoma medication number | 0.549 | 0.28525 | 1.056 | 0.0726 |

POAG: primary open-angle glaucoma, XFG: exfoliation glaucoma, SG: secondary glaucoma, LET: trabeculectomy, IOP: intraocular pressure
